# An Improved Ningxia Desert Herbaceous Plant Classification Algorithm Based on YOLOv8

**DOI:** 10.3390/s24123834

**Published:** 2024-06-13

**Authors:** Hongxing Ma, Tielei Sheng, Yun Ma, Jianping Gou

**Affiliations:** 1 School of Electrical Information Engineering, North Minzu University, Yinchuan 750021, China; mhx@nmu.edu.cn (H.M.); 20237402@stu.nmu.edu.cn (Y.M.); 2 School of Computer and Information Science, Southwest University, Chongqing 400715, China; cherish.gjp@gmail.com

**Keywords:** plant identification, YOLOv8, KernelWarehouse, spatial attention, dynamic detection head

## Abstract

Wild desert grasslands are characterized by diverse habitats, uneven plant distribution, similarities among plant class, and the presence of plant shadows. However, the existing models for detecting plant species in desert grasslands exhibit low precision, require a large number of parameters, and incur high computational cost, rendering them unsuitable for deployment in plant recognition scenarios within these environments. To address these challenges, this paper proposes a lightweight and fast plant species detection system, termed YOLOv8s-KDT, tailored for complex desert grassland environments. Firstly, the model introduces a dynamic convolutional KernelWarehouse method to reduce the dimensionality of convolutional kernels and increase their number, thus achieving a better balance between parameter efficiency and representation ability. Secondly, the model incorporates triplet attention into its feature extraction network, effectively capturing the relationship between channel and spatial position and enhancing the model’s feature extraction capabilities. Finally, the introduction of a dynamic detection head tackles the issue related to target detection head and attention non-uniformity, thus improving the representation of the target detection head while reducing computational cost. The experimental results demonstrate that the upgraded YOLOv8s-KDT model can rapidly and effectively identify desert grassland plants. Compared to the original model, FLOPs decreased by 50.8%, accuracy improved by 4.5%, and mAP increased by 5.6%. Currently, the YOLOv8s-KDT model is deployed in the mobile plant identification APP of Ningxia desert grassland and the fixed-point ecological information observation platform. It facilitates the investigation of desert grassland vegetation distribution across the entire Ningxia region as well as long-term observation and tracking of plant ecological information in specific areas, such as Dashuikeng, Huangji Field, and Hongsibu in Ningxia.

## 1. Introduction

Ningxia, located in northwest China, falls within arid and semi-arid regions characterized by a dry climate, scant precipitation, high evaporation rates, prolonged sunshine, significant diurnal temperature fluctuations, sandy winds, low soil fertility, and extensive land sandification [1]. Desert grassland [2] encompass more than 80% of Ningxia’s grassland expanse. Over recent years, intensified and more frequent droughts, attributed to global climate change, have exacerbated the aridity of desert grasslands. This has impeded plant growth, accelerated grassland vegetation degradation, compromised ecological functions, and diminished the stability and resilience of grassland ecosystems. Concurrently, escalated human activities, like overgrazing, indiscriminate cultivation, and mining, have also exerted substantial pressure on the desert grassland, leading to soil erosion, sand encroachment, and salinization and fostering conditions conducive to the proliferation of rodents, insects, poisonous plants, and diseases, culminating in biological disasters. These occurrences further exacerbate the deterioration of the grassland ecosystem, perpetuating a vicious circle. The progressive deterioration of the desert grassland ecosystems jeopardizes grassland protection and pastoral development.

Given these challenges, the swift and accurate identification of primary desert grassland plant species serves as a foundational step for monitoring changes in grassland community structure, delineating grassland classifications, assessing the current state of grassland degradation and restoration, and safeguarding grassland plant diversity. Presently, the identification of desert grassland vegetation species relies predominantly on visual and empirical assessments by trained professionals, necessitating a deep understanding of botany and the ability to discern plant categories based on characteristics such as leaf morphology, color, texture, and floral features [3]. However, this approach is time-consuming and complex and demands considerable expertise from species identifiers. Therefore, there is a pressing need for the development of machine-vision techniques and machine-learning algorithms to quickly and effectively detect plant species. With the advent of computer-vision technology, a plethora of machine-learning-based plant recognition algorithms already exist in the field, offering rapid and precise plant species identification capabilities.

Research on target detection in the field of deep learning has been more extensive in recent years [4,5,6], in which research on plant species detection has received widespread attention. Early research into plant recognition techniques comprised many stages. Firstly, image preprocessing emerged as a crucial step involving screening, color modification, quantity expansion, and deflation, aimed at improving the extraction of texture features from plant leaves. Secondly, feature detection and extraction were achieved using a specially constructed model designed to capture vital aspects of the plant. Finally, these features are encoded and classified using a classifier to yield the final recognition result [7]. Caglayan et al. [8] leveraged shape and color characteristics extracted from leaves in conjunction with k-nearest neighbors, support vector machines (SVMs), naive Bayes models, and random forest classification algorithms for plant-type classification. Satti et al. [9] proposed a system for plant detection utilizing digital image processing and machine-vision techniques, integrating artificial neural network (ANN) and Euclidean (KNN) classifiers. Sachin et al. [10] adopted an integrated local binary histogram gradient pattern image feature extraction approach for the detection and categorization of Indian crops. Jin et al. [11] introduced a novel method for automatic species identification using sparse representations of leaf tooth characteristics. However, this method has significant limitations, particularly in categorizing plants with subtle leaf shapes. Yu et al. [12] proposed a plant identification approach based on leaf contours and venation for categorization, albeit limited to species with evident contours and demonstrating weak generalization capacity. Li et al. [13] introduced an image segmentation approach based on the HSV model, addressing the issues of poor segmentation arising from noise in the standard image segmentation methods. Ding et al. [14] introduced a plant leaf image identification approach based on weighted local linear embedding and support vector machines, showing high classification precision. Liu et al. [15] combined texture features mixed with shape features and used a deep-belief network architecture as a classifier for plant leaf recognition. Wang et al. [16] developed a WPA-SVM succulent plant classification and recognition model, achieving favorable succulent plant classification results.

As an increasing number of researchers delve into the realm of computer vision, convolutional neural networks (CNNs), as a cornerstone of deep-learning models, have led to significant achievements in image recognition. Grinblat et al. [17] developed a relatively straightforward yet powerful neural network for the successful identification of three different bean species based on the morphological patterns of leaf veins. Pawara et al. [18] observed that CNNs demonstrate superior recognition precision and speed, particularly when fine-tuning AlexNet, as evidenced in comparative trials with GoogLeNet and classical conventional plant recognition methods. Sun et al. [19] introduced the BJFU plant dataset, which covers 10 plant classes, and devised an eight-layer, deep-learning model consisting of 26 residual component blocks, achieving a classification accuracy of 78.1%. Hu et al. [20] introduced a multi-scale fusion convolutional neural network surpassing the recognition precision of contemporaneous deep-learning models on the MK and Leafsnap datasets. Using the same dataset, Nhan et al. [21] proposed a scoring-based, multi-organ, image fusion model, which initially conducts single-organ image classification and then consolidates recognition results from multiple organs, demonstrating superior performance compared to single-organ recognition in plant species classification.

Gao et al. [22] curated a dataset comprising 293 natural grass plant images and developed an image recognition model of natural grass plants using the TF-slim module, fine-tuning parameters of the Inception V3 model through extensive experimentation. Bisen et al. [23] achieved 97% classification precision in the Swedish leaf dataset with a four-layer traditional convolutional neural network. Hussain et al. [24] leveraged a lightweight MobileNetV3 model with transfer learning, attaining a recognition precision of 91% on the Leafsnap dataset. Hassan et al. [25] employed CNNs and transfer-learning methods for identifying plant leaf diseases, resulting in improved performance in terms of precision and reduced training time. Arshed et al. [26] fine-tuned a ResNet50 model on a self-constructed dataset of grape, mango, and potato plant leaves, achieving an average recognition accuracy of 92.5%. Guang et al. [27] introduced a lightweight ECAENet recognition model, with its base network crafted via a neural architecture search approach, achieving a remarkable 99.75% recognition precision on the Flavia Leaf dataset.

While the aforementioned studies have obtained promising detection results, they have not explored desert grassland plants against intricate backgrounds [27]. When plant targets are occluded and blurred [24], effective recognition becomes challenging, often resulting in erroneous or missed detections. Moreover, many existing deep-learning-based plant detection algorithms enhance detection accuracy at the expense of increased computational complexity and larger model sizes [22], leading to slower detection speeds [26]. On the other hand, methods prioritizing low model computational complexity and swift detection speeds often sacrifice detection precision. Finding a balance between detection speed, precision, and model complexity in approaches for detecting plants in the Ningxia desert grasslands poses a formidable challenge, particularly considering the imperative for real-time performance during model deployment.

YOLOv8, the latest iteration in the YOLO family, distinguishes itself with high efficiency, accuracy, and a minimal model memory footprint. Leveraging YOLOv8 as a benchmark, we present a desert plant target recognition model, YOLOv8s-KDT, tailored for challenging environments. Our contributions are summarized as follows:

1. Dataset Construction: We curated a dataset for desert grassland plant detection in Ningxia, encompassing 30 classes of common desert grassland plants. Rigorous screening ensured the inclusion of only high-quality photos, which were then manually annotated to delineate desert grassland plant species.
2. Innovative Techniques: Our approach integrates the KernelWarehouse dynamic convolution technique, triple channel and spatial attention mechanisms, and a dynamic target detection head. These techniques effectively reduce model parameters, mitigate overfitting, and lower computational and memory demands. Moreover, our method significantly enhances the model’s ability to accurately recognize desert grassland plants.
3. YOLOv8s-KDT Method: We present the YOLOv8s-KDT method, which is lighter and more accurate than the original YOLOv8. With fewer parameters, reduced FLOPS, higher FPS, and easier deployment, it offers superior performance.
4. Mobile Application Development: We developed a mobile plant recognition app implementing the YOLOv8s-KDT algorithm, enabling real-time recognition of plant photos in Ningxia desert grassland.

## 2. Materials and Methods

### 2.1. Model Structure of the YOLOv8s Network

The YOLOv8 algorithm stands out for its exceptional balance between integrated detection precision and speed. Building upon the foundation of YOLOv5, YOLOv8 represents a single-stage target identification method that integrates state-of-the-art counting techniques. This iteration introduces a host of brand-new features and enhancements, markedly enhancing the model’s performance and adaptability. With improvements aimed at speed, accuracy, and usability, YOLOv8 offers a diverse range of models of different sizes, denoted as N/S/M/L/X scales, meticulously calibrated using scaling factors.

The YOLOv8 model network consists of three key components: a backbone network for feature extraction, a neck network, and a detecting head for feature fusion. The backbone network integrates elements of a cross-stage local network, optimizing processing efficiency and gradient enhancement. Furthermore, it incorporates the spatial pyramid pooling module, as observed in architectures like YOLOv5, to bolster spatial data extraction. Notably, the C3 module in YOLOv5 has been replaced by the lightweight C2f module in YOLOv8, enhancing adaptability to targets of varied sizes and shapes. The head component employs the current standard decoupled head structure, substantially reducing parameters and computational complexity while enhancing generalization and robustness. In summary, YOLOv8 represents a significant advancement over early iterations by introducing new features and enhancements.

However, despite its strengths, YOLOv8 exhibits limitations in recognizing complex backgrounds and displays reduced robustness when faced with occlusion and rotation. In this research application, there is a pressing need to enhance the model’s recognition precision while simultaneously improving its generalization ability, robustness, and inference speed.

### 2.2. KernelWarehouse Dynamic Convolution

**Figure 1:**
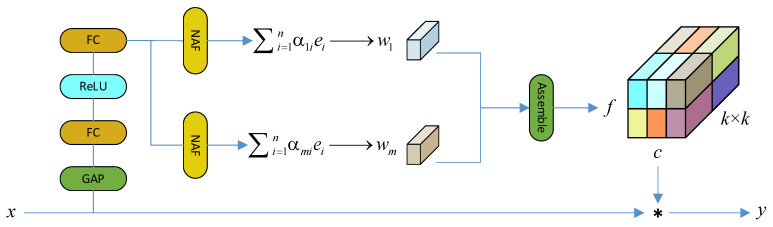
Schematic of the KernelWarehouse dynamic convolution method.

Some of the high-resolution plant photos included in the proposed dataset have complex backgrounds and characteristics that may not be readily discernible. However, the YOLOv8 model employs a single-feature extraction approach that overlooks the integration of multiple features. This reliance on a single convolutional neural network structure, devoid of consideration for parameter dependencies across various levels and adjacent layers, results in an excessive number of model parameters, heightened processing demands, and limited generalization capacity. To address these challenges and enhance both feature extraction and generalization abilities, we introduce the KernelWarehouse [28] technique to the backbone and neck of the network. The schematic diagram illustrating the KernelWarehouse technique is depicted in Figure 1.

KernelWarehouse is an extended form of dynamic convolution. Firstly, it entails the segmentation of the convolutional kernel into non-overlapping kernel units. Secondly, the linear mixing of each kernel unit is achieved utilizing a predefined repository shared across multiple adjacent convolutional layers. Lastly, the static convolutional kernels are replaced by sequential combinations of their respective blending results, thereby strengthening the convolutional parameter dependency between the same and succeeding layers by dividing and sharing the convolutional kernels. As a results, the model achieves heightened flexibility in design while substantially reducing mathematical parameters, thus facilitating easier implementation.

### 2.3. Triple Attention Mechanism

Attention mechanisms emulate human visual and cognitive systems, aiding models in focusing on essential sections of sequence data during processing, thereby boosting performance and generalization. Traditionally, enhancing attentional processes involves deepening the network to bolster the model’s comprehension of visual features. However, this approach often imposes computational burdens beyond the capacity of a lightweight network tailored for plant recognition. Given that the collected plant images boast a resolution of 3000 pixels × 4000 pixels, background information constitutes a significant portion, leading to the accumulation of redundant and invalid information during convolution iterations within a deep convolutional neural network. This accumulation hampers target information recognition precision. To increase the feature extraction capacity of the modified YOLOv8s-KDT for crucial details of desert grassland plants and mitigate the adverse effects of erroneous redundant information on plant recognition accuracy, we adopt an almost parameter-free, triple-attention mechanism [29].

**Figure 2:**
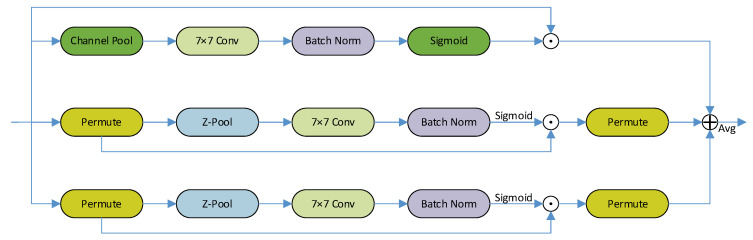
Structure of triplet attention.

Triplet attention is designed to establish dependencies between channel and spatial dimensions, thereby retaining rich information and structural details within the picture. This approach mitigates the risk of information loss or overfitting that can arise with the single-dimensional attention mechanism. Cross-dimensional interactions are accomplished by rotation operations and residual transformations, effectively reducing computational and memory requirements for lightweight networks. Channel and spatial attention are achieved through simple and efficient convolutional layers, enhancing the model’s capacity to distinguish desert grassland plants while maintaining fast identification speed. The structure of triplet attention is shown in Figure 2.

In YOLOv8s-KDT, the triplet attention mechanism can be integrated either after the backbone network’s C2f module or after the neck fusion network’s C2f module. However, empirical trials indicate superior performance when incorporating the triplet attention mechanism after the C2f module of the backbone network. This integration notably enhances the model’s recognition precision, while the addition of relatively few parameters significantly boosts the recognition speed.

### 2.4. Dynamic Target Detection Head

**Figure 3:**
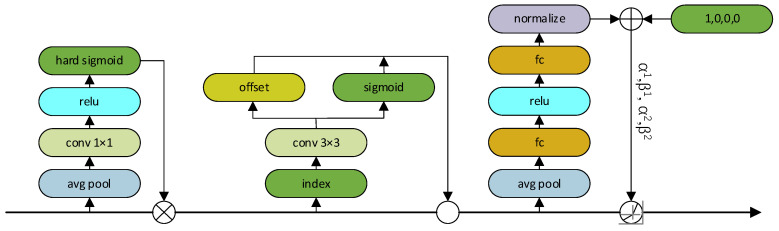
Structure of dynamic head.

Traditional detecting heads often require extensive training data and computational resources, leading to challenges such as overfitting or memory constraints. Convergence speed tends to be slow, and the model’s generalization ability may be lacking. In the original YOLOv8 model, the detection head relies on an anchor-based (Anchhor) technique to predefine target categories and positions, which can result in information loss, overfitting, and difficulties in identifying targets. Furthermore, the static allocation technique used in the YOLOv8 detecting head may overlook certain samples or encounter issues with double counting. In contrast, DyHead [30] employs an attention method to integrate multiple target detection heads. By leveraging attention across feature hierarchies for scale perception, spatial locations for spatial perception, and output channels for task perception, Dyhead significantly enhances the expressive power of the modeled target detection head without increasing the computational complexity. The architecture of the dynamic detecting head is shown in Figure 3.

The attention function is converted into three sequential attentions, each focusing on only one dimension:

(1) $$W\left(F\right)=\pi_{C}\left(\pi_{S}\left(\pi_{L}\left(F\right)\cdot F\right)\cdot F\right)\cdot F$$

where $\pi_{C}$ is the channel attention, $\pi_{S}$ is the spatial perceptual attention, and $\pi_{L}$ is the scaling perceived attention. DyHead requires only a minimal number of operations on input features without the need for constructing additional complex network structures. Furthermore, DyHead possesses the capability to adaptively adjust the feature fusion method based on various tasks and datasets. This adaptability enhances the detection head’s attention to crucial information regarding desert steppe plants, including categories, locations, sizes, shapes, and other relevant aspects. As a result, DyHead not only improves the model’s detection precision, resilience, and generalization capacity but also minimizes computing and memory consumption.

### 2.5. Model Structure of the YOLOv8s-KDT Network

**Figure 4:**
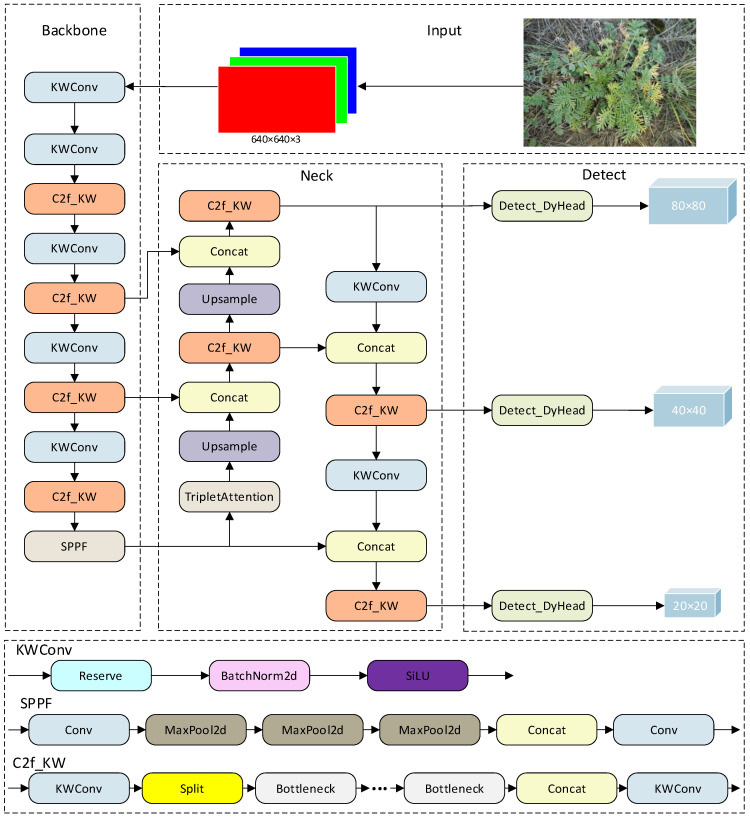
Structure of YOLOv8s-KDT model.

The improved network model is named YOLOv8s-KDT, and the model structure is shown in Figure 4.

In Figure 4, YOLOv8s-KDT is separated into three core components: the backbone, responsible for feature extraction on the input plant photos; the neck, facilitating feature fusion across the collected feature maps; and the head, executing regression prediction. Firstly, YOLOv8s-KDT integrates the dynamic convolution technique KernelWarehouse within the backbone feature extraction network. This method enables adaptive adjustments to the size of the “warehouse” and the dimensions and quantity of sub-kernels, according to the desert grassland plant dataset. By reducing model parameters and computational load while maintaining high accuracy, KernelWarehouse enhances reasoning efficiency, facilitating model deployment. Secondly, the model incorporates the almost parameter-free, triple-attention mechanism, efficiently capturing the interactions between different dimensions in the desert grassland plant images without increasing the network depth. This augmentation improves the feature extraction and generalization capabilities while greatly improving recognition speed. Finally, the inclusion of the DyHead dynamic detection head unifies the detection head and attention without introducing any computational cost. This integration significantly enhances the identification power of the target detection head for desert grassland plants.

## 3. Datasets and Experimental Environments

### 3.1. Plant Datasets

Plant species detection plays a pivotal role in identifying, categorizing, and detecting plant instances within specific regions; finding applications in ecological protection; landscaping; and agricultural production. However, the effectiveness of plant species detection relies heavily on representative and diverse datasets, considering the variations in plant growth state (e.g., different growth stages, different postures), leaf shape, flower shape, and fruit shape, among other factors, encountered in real-world environments. Additionally, the influence of environmental factors, like light, angle, and occlusion, further complicates the task. To address these challenges and enhance precision and resilience, powerful deep-learning algorithms are employed to process and evaluate the data.

Existing plant species detection datasets suffer from several shortcomings, including a limited variety of species, a relatively small quantity of data, and the absence of complex background conditions encountered in natural environments. Furthermore, the majority of these datasets are derived from images of plant samples collected in laboratory settings, resulting in a lack of diversity in scenes that fail to adequately reflect the model’s generalization capacity and resilience. Given that Ningxia experiences a temperate continental dry and semi-arid climate characterized by low and relatively concentrated precipitation, abundant light energy, high evaporation rate, and vegetation dominated by sandy plants with thin leaves, it is challenging to extract meaningful information solely from harvested leaves. Consequently, the complete plant is included in the plant dataset. However, due to similarities in background and features of the desert grassland plants in their natural habitat, distinguishing features are often indiscernible, impeding effective identification and analysis. Images of desert grassland plants were taken between October 2022 and December 2023 using a Canon EOS R6 camera (Canon, Tokyo, Japan), and the image resolution was set to 3000 pixels × 4000 pixels in order to enrich the diversity of plant data, improve the generalization ability of recognition models, and capture natural image data from different angles under different lighting conditions.

**Figure 5:**
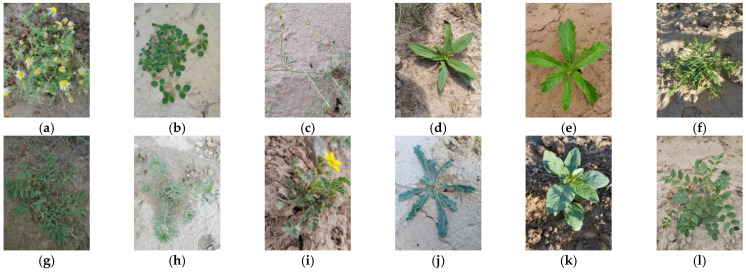
Plant images: (**a**) *Aster altaicus*; (**b**) *Medicago archiducis-nicolaii*; (**c**) *Astragalus melilotoides*; (**d**) *Plantago depressa*; (**e**) *Dracocephalum peregrinum*; (**f**) *Cynanchum thesioides*; (**g**) *Potentilla multicaulis*; (**h**) *Peganum multisectum*; (**i**) *Sibbaldianthe bifurca*; (**j**) *Limonium bicolor*; (**k**) *Amaranthus retroflexus*; (**l**) *Glycyrrhiza uralensis*.

**Figure 6:**
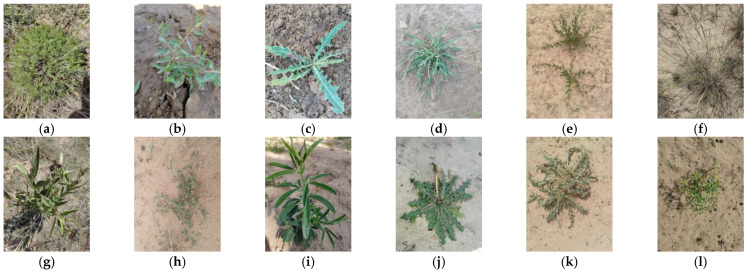
Plant images: (**a**) *Artemisia gansuensis*; (**b**) *Lycium chinense*; (**c**) *Bitter bitter cabbage*; (**d**) *Bitter bitter cabbage and milk lettuce*; (**e**) *Cynanchum komarovii Al*; (**f**) *Agropyron mongolicum Keng*; (**g**) *Cynanchum hancockianum*; (**h**) *Lespedeza daurica*; (**i**) *Thermopsis lanceolata*; (**j**) *Taraxacum mongolicum Hand*; (**k**) *Olgaea leucophylla*; (**l**) *Euphorbia kozlovii*.

**Figure 7:**
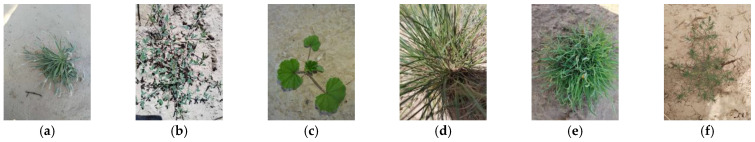
Plant images: (**a**) *Plantago minuta*; (**b**) *Lespedeza davurica*; (**c**) *Malva pusilla*; (**d**) *Stipa bungeana*; (**e**) *Ixeridium chinense*; (**f**) *Salsola collina*.

A total of 3300 images of 30 common desert grassland plants were predominantly sourced from Mahuang Mountain and Dashukeng in Yanchi County, Ningxia, as well as Luoshan in Tongxin County. Figure 5, Figure 6 and Figure 7 show the sample images of 30 plant types. Using the ImgAug library in Python, the dataset underwent augmentation, resulting in 6600 images, amounting to 3.71 GB. Following data cleaning to address issues such as fuzzy captures and obscured branches and leaves, the dataset comprised 30 classes of plant image data totaling 6504 images and 3.52 GB in size. Among these, the distribution of images per plant species is as follows: 184 images of *Medicago archiducis-nicolaii*, 14 images of *Bitter bitter cabbage and milk lettuce*, 266 images of *Bitter bitter cabbage*, 208 images of *Stipa bungeana*, 330 images of *Astragalus melilotoides*, 264 images of *Plantago depressa*, 298 images of *Lespedeza davurica*, 268 images of *Dracocephalum peregrinum*, 234 images of *Cynanchum thesioides*, 204 images of *Amaranthus retroflexus*, 184 images of *Sibbaldianthe bifurca*, 196 images of *Limonium bicolor*, 280 images of *Thermopsis lanceolata*, 344 images of *Glycyrrhiza uralensis*, 314 images of *Taraxacum mongolicum Hand*, 214 images of *Plantago minuta*, 196 images of *Malva pusilla*, 208 images of *Salsola collina*, 228 images of *Aster altaicus*, 260 images of *Lycium chinense*, 174 images of *Ixeridium chinense*, 260 images of *Potentilla multicaulis*, 316 images of *Peganum multisectum*, 352 images of *Euphorbia kozlovii*, 134 images of *Cynanchum komarovii Al*, 206 images of *Olgaea leucophylla*, 178 images of *Lespedeza daurica*, 100 images of *Artemisia gansuensis*, 58 images of *Agropyron mongolicum Keng*, and 32 images of *Cynanchum hancockianum*.

### 3.2. Data Enhancement

**Figure 8:**
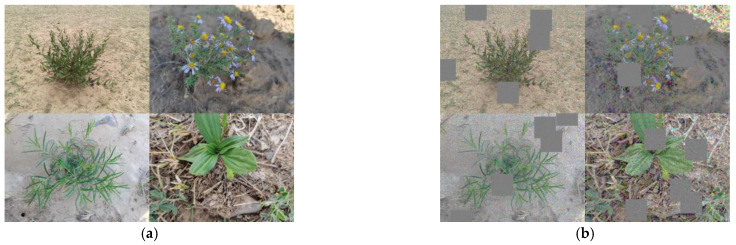
Data enhancement: (**a**) original data, (**b**) enhanced data.

To enhance the precision and robustness of the plant species recognition model and to mitigate potential overfitting issues for improved practical application, data augmentation techniques are employed to expand the dataset. Python’s ImgAug library is a versatile tool for image enhancement, offering a wide range of techniques with configurable parameters, efficient processing speed, and seamless integration. The ImgAug tool is used to apply random masking blocks, Gaussian blur, and mosaics effect to the gathered plant photos, thus enlarging the dataset. These augmentation techniques effectively enhance the richness and diversity of the Ningxia desert grassland plant dataset, improving the model’s adaptability to various plant morphologies and environmental conditions, thus making the trained models more reliable and accurate in prediction tasks. To illustrate the effects of data augmentation, four plant photos are selected to showcase the transformations before and after augmentation, as depicted in Figure 8.

### 3.3. Data Labeling

In order to enhance the precision of the output of the target detection model and enable the network model to learn plant attributes and position information, it is essential to annotate the plants in the image. The annotation process is carried out using the LabelImg-1.8.6 tool to create a dataset in the VOC format. The resulting labeled XML file contains detailed information about the plant’s name and location within the image. Both the annotated image and their corresponding labeled files are utilized for model training and validation purposes.

### 3.4. Experimental Environment and Hyperparameter Configuration

All models were trained on a Ubuntu 22.04 server equipped with an Intel (R) Corei9-14900K CPU and a RTX4090 GPU. The model training environment was PyTorch 2.1.0, Python 3.9, and Cuda 12.1. Training was conducted over 200 rounds with a batch size of 16 and images resized to 640 pixels × 640 pixels. The initialized learning rate was set to 0.01, and no pre-training weights were utilized, while other parameters retained the original default values. The dataset comprised a total of 6504 plant photos, randomly divided into training, validation, and test sets in a 6:2:2 ratio. Specifically, the training set contains 3902 images, the validation set contains 1301 images, and the test set contains 1301 images.

### 3.5. Model Evaluation Metrics

Evaluating a model’s performance is critical because it provides insights into its effectiveness. Various evaluation metrics offer different perspectives on the model’s performance. Metrics such as recall, precision, average precision mean, and F1 score, are commonly used to assess a model’s detection capacity. Additionally, metrics like frames per second (FPS) and floating-point operations (FLOPS) are used to evaluate the model’s detection speed and computational complexity. In this study, precision is employed as a key metric to measure the efficacy of the model. Precision represents the fraction of true positive predictions (TP) among all instances predicted as positive (TP + FP). It is calculated using the following formula:

(2) $$P(\mathrm{Precision})=\frac{TP}{TP+FP}$$

Recall measures the proportion of true positive predictions (TP) that are correctly identified by the model out of all actual positive samples, i.e., TP + FN. A higher recall rate indicates better performance in identifying positive classes. Recall is mathematically defined as follows:

(3) $$R(\mathrm{Recall})=\frac{TP}{TP+FN}$$

The F1 score serves as the harmonic mean of precision and recall, offering a balanced assessment of both metrics. A higher F1 score indicates superior performance in terms of both precision and recall. The F1 score is calculated using the following formula:

(4) $$F_{1}=2\times \frac{\mathrm{Precision}\times \mathrm{Recall}}{\mathrm{Precision}+\mathrm{Recall}}$$

Average precision (AP) is calculated as follows:

(5) $$AP=\int_{0}^{1}P(R)dR$$

The mAP is calculated as the arithmetic mean of the average precision (AP) values achieved for each category within the dataset. this metric is used to evaluate the overall model performance. The mAP is defined as shown in the following formula:

(6) $$mAP=\frac{1}{N}\sum_{i=1}^{N}AP_{i}$$

FLOPs refer to the number of floating-point operations required for the model execution. They serve as a fundamental metric for gauging the computational complexity of a model. Floating-point operations are mathematical calculations using actual numbers with fractional portions, and they play a key part in different computational tasks in CNNs, including convolution, matrix multiplication, element-by-element operations, and nonlinear activation. The use of floating-point operations is essential to facilitate the efficient processing and manipulation of numerical information within the CNN, and these processes enable precise and complex computations, which must be performed when performing a series of operations such as image classification, object recognition, and image synthesis. FPS denotes the rate at which the model can process image frames. It stands as a crucial metric in assessing the operational speed of a target identification algorithm. A higher FPS signifies the algorithm’s capability to analyze a larger number of images within a given time frame, thus indicating faster detection speed.

## 4. Results and Discussion

### 4.1. Recognition Results

The enhanced YOLOv8s-KDT model was employed to identify a total of 30 plant classes in the photos of Ningxia desert grassland. Figure 8 shows some of the plant recognition results.

**Figure 9:**
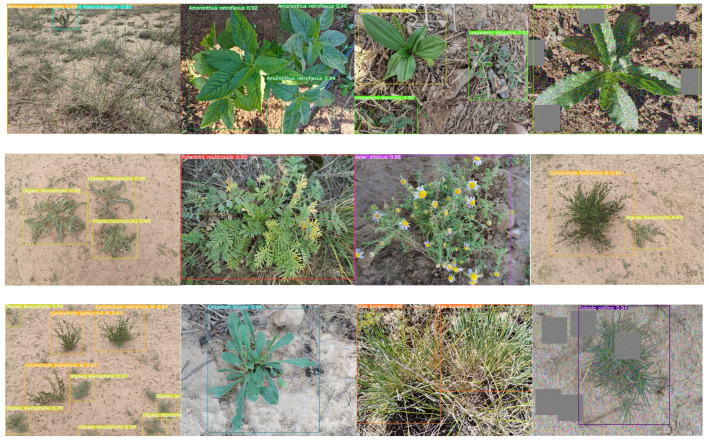
Grassland plant image recognition results.

As shown in Figure 9, the improved YOLOv8s-KDT model can reliably identify plant photos that have more than one target. It can also identify photos of plants that are partially obscured or have been processed with Gaussian blur. We can also observe in Figure 9 that the recognition accuracy is higher when the camera is closer to the shooting plant. When the camera is far away from the shooting plant, although the recognition accuracy is low, the target plant can still be accurately recognized, and our improved model performs well.

### 4.2. Test Results on Plant Dataset

**Figure 10:**
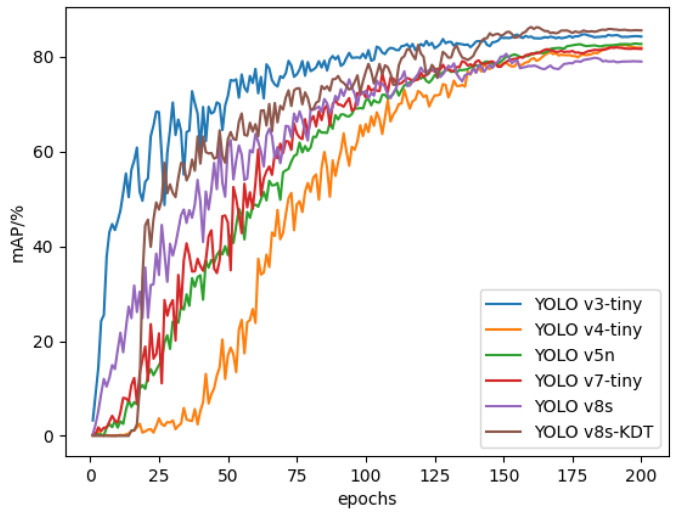
Variation curves of mAP for different models.

To evaluate the performance of the upgraded YOLOv8s-KDT model, we compared it with several other target detection models: YOLOv3-tiny, YOLOv4-tiny [31], YOLOv5n [32], YOLOv7-tiny [33,34], and YOLOv8s. This assessment was conducted using our custom desert grassland plant dataset under identical experimental conditions. Figure 10 illustrates the fluctuation in the mean average precision (mAP) training curve across the enhanced model and the aforementioned models.

Figure 10 clearly demonstrates that the enhanced YOLOv8s-KDT model surpasses YOLOv3-tiny, YOLOv4-tiny, YOLOv5n, YOLOv7-tiny, and YOLOv8s in terms of mean average precision (mAP). Despite YOLOv8s-KDT exhibiting a marginally slower convergence speed compared to YOLOv3-tiny, it achieves superior convergence and a higher mAP after 200 rounds of sufficient training.

To illustrate the precision and performance of the upgraded YOLOv8s-KDT model more effectively, Table 1 displays the experimental results comparing the recognition accuracy and performance of YOLOv8s-KDT with other models.

Table 1 provides a clear insight into the precision comparison between YOLOv7-tiny and YOLOv8s-KDT in detecting desert grassland plants. Although YOLOv7-tiny exhibits only a 0.5 percentage point difference in precision compared to YOLOv8s-KDT, its recall, mAP, F1 score, and FPS fall short of the efficiency achieved by YOLOv8s-KDT. Specifically, YOLOv8s-KDT achieves a recall of 81.6% and an mAP of 86.6%. This study aims to develop models with heightened precision and faster detection capabilities, necessitating a consideration of metrics such as detection speed and recognition accuracy. Despite YOLOv3-tiny boasting an FPS of 566 frames/s, a mere 22 frames/s lower than YOLOv8s-KDT, it lags behind in terms of accuracy, recall, mAP, and F1 score compared to the latter. Overall, the upgraded YOLOv8s-KDT model demonstrates superior performance over YOLOv3-tiny, YOLOv4-tiny, YOLOv5n, YOLOv7-tiny, and YOLOv8s. The data labeled using underlines in Table 1 are the second-best results for that column, and the data in parentheses represent how much YOLOv8s-KDT changed compared to the second-best model. Upward arrows represent increased results.

### 4.3. Test Results on Public Datasets

To enhance the objectivity of evaluating the proposed model in this paper, we selected the public dataset PASCAL VOC07 + 12, which comprises a total of 20 targets. The dataset was randomly divided into three parts in a 7:2:1 ratio within the same experimental environment. YOLOv8s-KDT was then tested and compared with YOLOv3-tiny, YOLOv4-tiny, YOLOv5n, YOLOv7-tiny, and YOLOv8s. The comparison results are presented in Table 2.

From Table 2, it is evident that YOLOv4-tiny exhibits the highest precision among the models compared, with its mAP and F1 scores also surpassing those of YOLOv8s-KDT by 0.4 and 1.6 percentage points, respectively. However, its FLOPs are 6.8G higher than those of YOLOv8s-KDT. While YOLOv5n boasts a lower FLOP count of 4.2G, it lags behind YOLOv8s-KDT by 4.9%, 2.9%, 5%, and 3.8% in precision, recall, mAP, and F1 scores, respectively, indicating inferior performance in identifying the PASCAL VOC07 + 12 public dataset. Both YOLOv3-tiny and YOLOv7-tiny feature slightly lower FLOPs than the upgraded YOLOv8s-KDT model. However, they also exhibit lower accuracy, recall, mAP, and F1 scores compared to YOLOv8s-KDT. While YOLOv8s demonstrates higher precision than the upgraded YOLOv8s-KDT model, its recall, mAP, and F1 scores are lower, and its FLOPs are 28.5G, more than double that of YOLOv8s-KDT. The collective assessment of identification accuracy, recall, F1 scores, and FLOPs indicates that the enhanced model, YOLOv8s-KDT, delivers superior detection performance on the public dataset PASCAL VOC07 + 12.

### 4.4. Ablation Experiment

To assess the effectiveness of the enhanced YOLOv8s-KDT model, conducting comparative ablation experiments is crucial. This study conducted eight ablation experiments to evaluate model performance consistently utilizing the same dataset, training setup, and methodology. Validation analyses encompassed precision, recall, combined evaluation metrics, and mAP.

The initial row of Table 3 presents the training outcomes of the original YOLOv8s network without any enhancement strategies. Compared to the baseline YOLOv8s model, the incorporation of KernelWarehouse dynamic convolution, dynamic detection header, or the integration of the triplet attention method results in improved detection rates, enhanced precision, and reduced FLOPS across all new models. Specifically, when employing the KernelWarehouse method, FLOPS decrease by approximately 50%. Additionally, mAP is augmented by 3.5% and 1.7% with the KernelWarehouse method and DyHead, respectively. The triplet attention method boosts the recall rate by 1.0%. Following three distinct algorithm enhancements, the culmination is the YOLOv8s-KDT model. In comparison to the original YOLOv8s, we observe significant improvements of 4.3%, 5.8%, 5.1%, and 5.6% in precision, recall, F1 score, and mAP, respectively. Moreover, it boasts the lowest FLOPs and an FPS exceeding 580. In summary, the enhanced YOLOv8s-KDT model achieves a recall of 81.6% and an mAP of 86.6%. Its detection speed is significantly bolstered by the reduction in model parameters and FLOPS coupled with an increase in FPS, rendering it suitable for deployment across both mobile and fixed-observation platforms.

**Figure 11:**
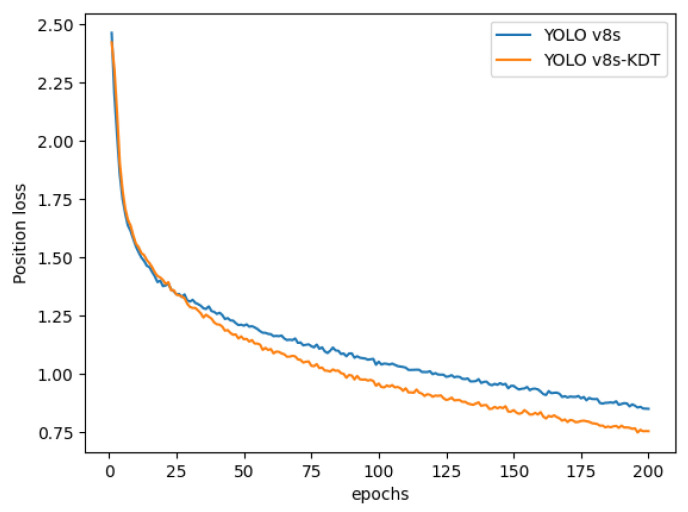
Position loss curve.

For a deeper analysis of the model’s performance before and after the improvement, we extracted the position loss values of YOLOv8s-KDT and YOLOv8s following 200 iterations, as shown in Figure 11.

As shown Figure 11, the enhanced YOLOv8s-KDT model demonstrates faster convergence and a significantly higher convergence rate compared to YOLOv8s. This improvement can be attributed to the incorporation of the KernelWarehouse and triplet attention methods.

**Figure 12:**
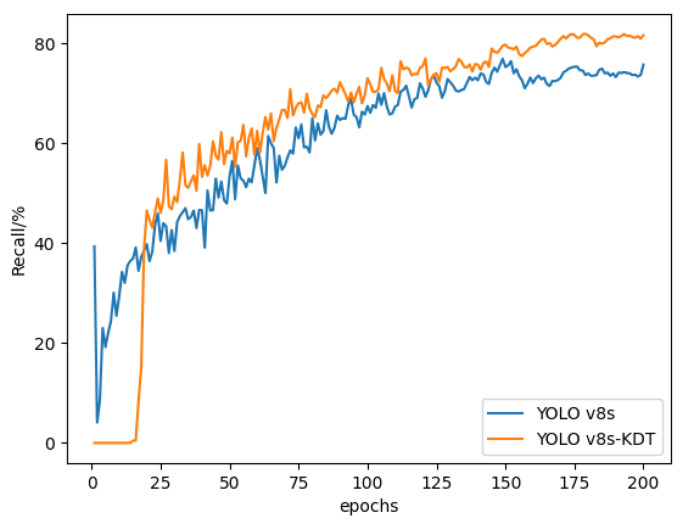
Recall rate curve.

The recall curves of the models are presented in Figure 12. The graphs illustrate a consistent increase in recall as the number of training rounds increases for both algorithms. However, beyond the 150-epoch, a notable divergence is observed between the enhanced YOLOv8s-KDT model and the original YOLOv8s model.

**Figure 13:**
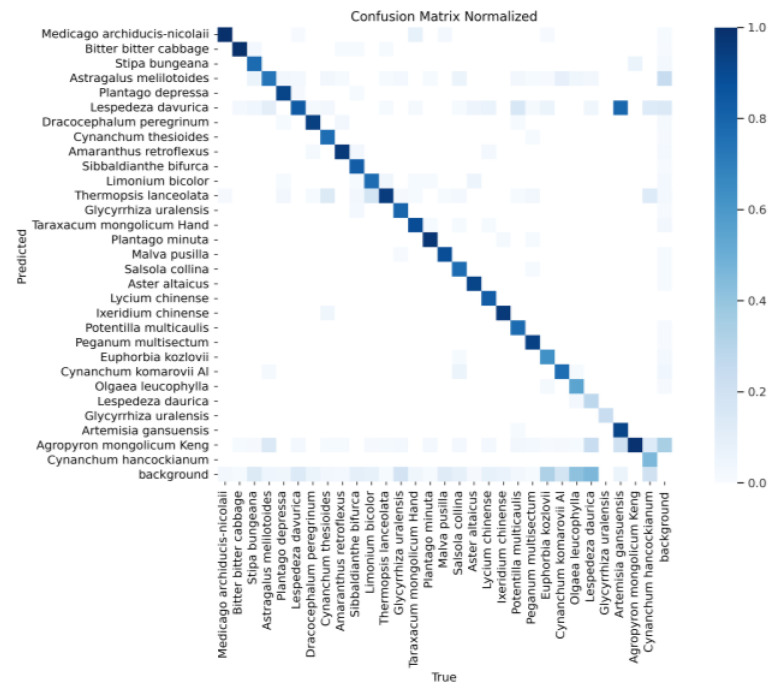
Confusion matrix of YOLOv8s.

**Figure 14:**
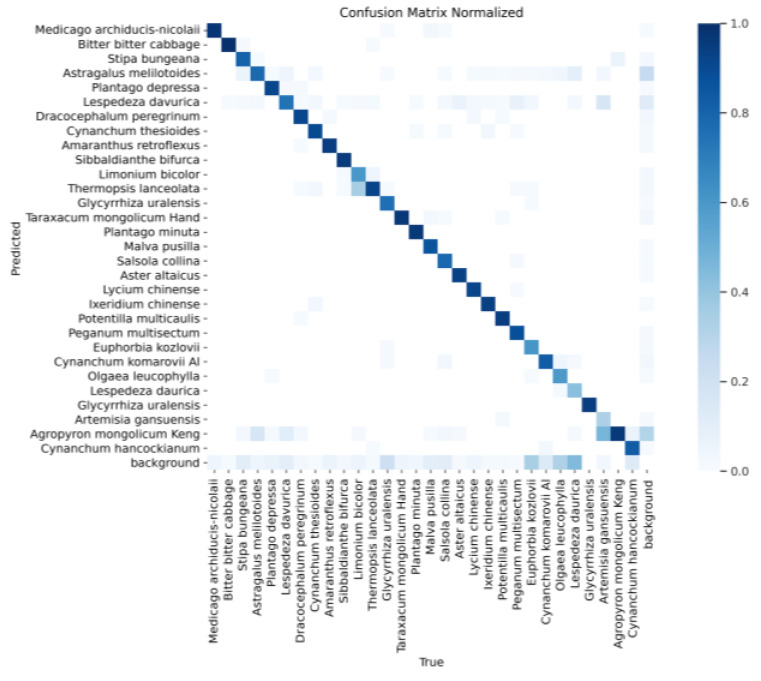
Confusion matrix of YOLOv8s-KDT.

Figure 13 shows the confusion matrix of YOLOv8s, and Figure 14 shows the confusion matrix of the YOLOv8s-KDT model. These two confusion matrices indicate that the original YOLOv8s model recognizes Artemisia gansuensis as Lespedeza davurica, and the confusion matrix indicates that the original YOLOv8s has a high false detection rate. In contrast, the YOLOv8s-KDT model proposed in this paper significantly improves the recognition accuracy of Artemisia gansuensis, and the false detection rate is reduced accordingly.

**Figure 15:**
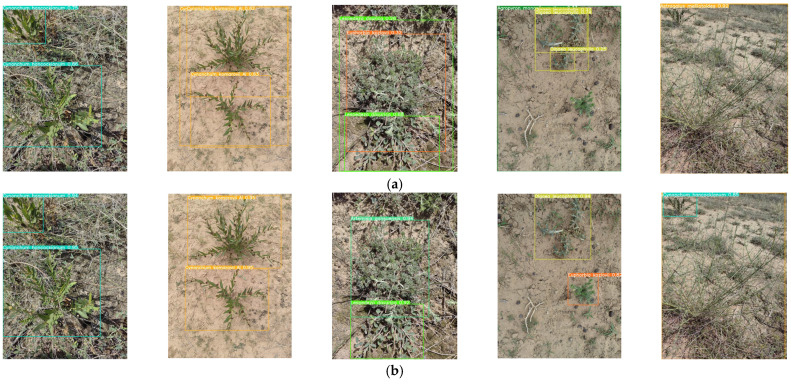
(**a**) YOLOv8s original model detection results graph; (**b**) YOLOv8s-KDT test result graph.

Figure 15 shows more intuitively the effect of the improved YOLOv8s-KDT model and the original YOLOv8s model on plant detection in the complex background of desert grassland.

## 5. Application

To support the study of desert grassland plant species and their distribution across the Ningixa region as well as to monitor plant growth patterns utilizing the enhanced YOLOv8s-KDT model, we designed and developed a mobile plant image recognition app. Additionally, we combined the plant recognition model with the fixed-point ecological information observation platform for remote tracking purposes.

**Figure 16:**
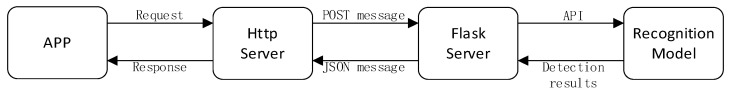
Flowchart of plant image recognition realization.

The plant image identification app is designed using the uni-app framework and packaged and distributed through the Android Studio tool. To facilitate future model updates, we have employed the Flask front-end framework to deploy the plant identification model on the AliCloud server. The process of plant identification APP recognition is shown in Figure 16.

**Figure 17:**
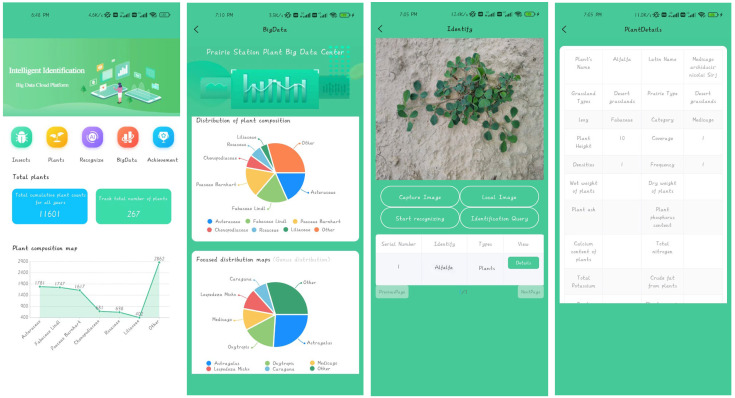
Plant image recognition APP interface.

The key functions of the APP are as follows: ① Image Upload and Identification: Users can either take new photos or select plant images from the gallery within the APP. The selected images are then uploaded to the cloud server, where YOLOv8s-KDT is called to automatically identify the images. The identification results are swiftly returned to the client, typically within just 1 s over a standard 4G network. Following the identification, users can assess comprehensive information about the plant, including details such as grassland type, density, nitrogen, phosphorus, and potassium content, wet and dry weight, as well as the geographical coordinates of the plant’s distribution. ② Database Contribution: Users have the option to contribute photographs to the database directly through the APP. This functionality aids in expanding the original plant dataset and enriches the variety of plants that the model can identify, thereby benefiting researchers. The user interface for each module of the plant image recognition App is shown in Figure 17.

**Figure 18:**
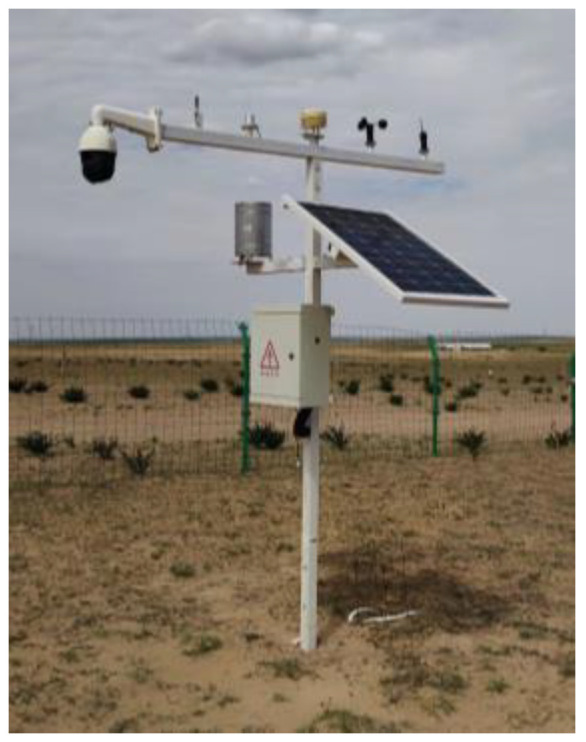
Targeted ecological information observation platform in kind.

With STM32 as the control core, the fixed-point ecological information observation platform can accomplish real-time monitoring and tracking of more than 10 types of ecological environment data, such as light intensity, rainfall, air temperature and humidity, soil nitrogen, phosphorus, potassium, and other major elements, and at the same time, it can conduct real-time collection and tracking of plant image data at the observation point. The physical object of the fixed-point ecological information observation platform is shown in Figure 18.

## 6. Conclusions

In conclusion, we presented a robust desert steppe vegetation recognition model based on YOLOv8s architecture and integrated it on a fixed-point plant observation platform. Through extensive experimentation and rigorous evaluation, we have demonstrated the effectiveness of our proposed model in accurately and swiftly identifying desert steppe plants in complex environmental backgrounds. The key improvements made to achieve this objective include the following:

1. Development of a comprehensive dataset comprising 30 types of desert steppe plants, ensuring the suitability of our proposed algorithm for deployment on field environment surveillance platforms.
2. Addressing challenges such as varied samples of desert grassland plants in Ningxia, complex backgrounds, and low recognition accuracy inherent in the YOLO series of detection algorithms. We propose the YOLOv8s-KDT model, equipped with the KernelWarehouse dynamic convolution and triplet attention mechanism to enhance the feature extraction network. Additionally, the model integrates a dynamic detection head to significantly enhance recognition capabilities and speed for desert grassland plats.
3. The enhanced YOLOv8s-KDT model achieves impressive performance metrics for desert grassland plant recognition, with a precision (P) of 82.8%, recall (R) of 81.6%, F1 score of 82.2%, mean average precision (mAP) of 86.6%, FLOPs of 14.0G, and FPS of 588 frames/sec. Compared to other YOLO models, YOLOv8s-KDT demonstrates superior accuracy and speed in distinguishing common plants in desert grassland environments, making it more suitable for deployment in mobile applications.
4. In practical applications, we have developed a mobile plant identification app and a fixed-point plant ecological information observation platform. These tools enable mobile identification of plant images in Ningxia desert grassland and real-time monitoring and long-term tracking of ecological information at observation points.

The current dataset is not rich enough, and the number of images in the dataset is small. The memory footprint of the improved model is not small enough to make it difficult to deploy the model in some cases where computational resources are extremely limited.

In our future research, we intend to expand the existing dataset by collecting more images of different types of desert grassland plants. This will contribute to improving the model’s generalizability and stability. In addition, we will investigate significantly reducing the model’s memory footprint size without compromising accuracy to ensure that it can be better deployed to run in environments with severely limited computational resources for real-time detection.

## Figures and Tables

**Table 1:** Comparison of network model recognition accuracy and performance.

Models	P/%	R/%	mAP/%	F1/%	FPS
YOLOv3-tiny	78.8	79.2	82.9	79.0	566
YOLOv4-tiny	81.6	75.0	82.4	78.2	238
YOLOv5n	79.8	74.4	82.7	77.0	323
YOLOv7-tiny	82.3	73.4	81.7	77.6	358
YOLOv8s	78.3	75.8	81.0	77.0	535
YOLOv8s-KDT	82.8 (0.5↑)	81.6 (2.4↑)	86.6 (3.7↑)	82.2 (3.2↑)	588 (22↑)

**Table 2:** Network model VOC dataset performance test.

Models	P/%	R/%	mAP/%	F1/%	FLOPs
YOLOv3-tiny	60.8	51.5	54.2	55.8	13.0
YOLOv4-tiny	81.2	62.3	70.0	70.5	20.7
YOLOv5n	70.8	60.3	65.1	65.1	4.2
YOLOv7-tiny	70.8	63.9	67.2	67.2	13.3
YOLOv8s	77.6	61.1	69.2	68.4	28.5
YOLOv8s-KDT	75.7	63.2	70.1	68.9	13.9

**Table 3:** Network model ablation experiments.

Models	Based Models	KW	DyHead	TA	P/%	R/%	F1/%	mAP/%	FLOPs (G)	FPS
Model 1	YOLOv8s				78.3	75.8	77.0	81.0	28.5	535
Model 2	YOLOv8s			√	77.9	76.8	77.3	80.2	28.5	865
Model 3	YOLOv8s		√		78.3	77.6	77.9	82.7	28.2	657
Model 4	YOLOv8s	√			83.9	80.1	81.9	84.5	14.3	493
Model 5	YOLOv8s		√	√	78.4	75.2	76.7	80.8	28.2	649
Model 6	YOLOv8s	√	√		79.6	78.1	78.8	84.1	14.0	514
Model 7	YOLOv8s	√		√	83.6	80.3	81.9	86.4	14.3	541
Model 8	YOLOv8s	√	√	√	82.8	81.6	82.1	86.6	14.0	588

## Data Availability

The data presented in this study can be requested from the corresponding author, and these data are not currently available for public access.

## References

[1] Xie J., Lu Z., Xiao S., Yan C. (2023). The Latest Desertification Process and Its Driving Force in Alxa League from 2000 to 2020. Remote Sens..

[2] Zhao J., Shi C., Wang L., Han X., Zhu Y., Liu J., Yang X. (2023). Functional Trait Responses of *Sophora Alopecuroides* L. Seedlings to Diverse Environmental Stresses in the Desert Steppe of Ningxia, China. Plants.

[3] Bhagat M., Kumar D. (2022). A Comprehensive Survey on Leaf Disease Identification & Classification. Multimed. Tools Appl..

[4] Bello R.-W., Oladipo M. (2024). Mask YOLOv7-Based Drone Vision System for Automated Cattle Detection and Counting. Artif. Intell. Appl..

[5] Chen M., Chen Z., Luo L., Tang Y., Cheng J., Wei H., Wang J. (2024). Dynamic Visual Servo Control Methods for Continuous Operation of a Fruit Harvesting Robot Working throughout an Orchard. Comput. Electron. Agric..

[6] Remmelzwaal L. (2023). Object Detection and Tracking for Crate and Bottle Identification in a Bottling Plant Using Deep Learning. Artif. Intell. Appl..

[7] Allaoua Chelloug S., Alkanhel R., Muthanna M.S.A., Aziz A., Muthanna A. (2023). MULTINET: A Multi-Agent DRL and EfficientNet Assisted Framework for 3D Plant Leaf Disease Identification and Severity Quantification. IEEE Access.

[8] Caglayan A., Guclu O., Can A. (2013). A Plant Recognition Approach Using Shape and Color Features in Leaf Images. Image Analysis and Processing–ICIAP 2013, Proceedings of the 17th International Conference, Naples, Italy, 9–13 September 2013.

[9] Satti V., Satya A., Sharma S. (2013). An Automatic Leaf Recognition System for Plant Identification Using Machine Vision Technology. Int. J. Eng. Sci. Technol. (IJEST).

[10] Jadhav S.B., Patil S.B. (2024). Plant Leaf Species Identification Using LBHPG Feature Extraction and Machine Learning Classifier Technique. Soft Comput..

[11] Jin T., Hou X., Li P., Zhou F. (2015). A Novel Method of Automatic Plant Species Identification Using Sparse Representation of Leaf Tooth Features. PLoS ONE.

[12] Yu X., Xiong S., Gao Y., Zhao Y., Yuan X. Multiscale Crossing Representation Using Combined Feature of Contour and Venation for Leaf Image Identification. Proceedings of the 2016 International Conference on Digital Image Computing: Techniques and Applications (DICTA).

[13] Li C., Zhang Y., Wang C. (2020). Research on HSV model for identifying and locating round plants. Comput. Eng. Appl..

[14] Ding J., Liang D., Yan Q. (2013). Recognition method of plant leaves based on WLLE and SVM. J. Anhui Univ..

[15] Liu N., Kan J.-M. (2016). Plant leaf identification based on the multi-feature fusion and deep belief networks method. J. Beijing For. Univ..

[16] Wang S., Meng Z., He L., Yang J. (2020). Classification and Identification of Succulent Plants Based on WPA-SVM. Microcomput. Appl..

[17] Grinblat G.L., Uzal L.C., Larese M.G., Granitto P.M. (2016). Deep Learning for Plant Identification Using Vein Morphological Patterns. Comput. Electron. Agric..

[18] Pawara P., Okafor E., Surinta O., Schomaker L., Wiering M. (2017). Comparing Local Descriptors and Bags of Visual Words to Deep Convolutional Neural Networks for Plant Recognition. Proceedings of the 6th International Conference on Pattern Recognition Applications and Methods.

[19] Sun Y., Liu Y., Wang G., Zhang H. (2017). Deep Learning for Plant Identification in Natural Environment. Comput. Intell. Neurosci..

[20] Hu J., Chen Z., Yang M., Zhang R., Cui Y. (2018). A Multiscale Fusion Convolutional Neural Network for Plant Leaf Recognition. IEEE Signal Process. Lett..

[21] Nhan N.T.T., Binh D.T., Hoang N.H., Hai V., Hai T.T.T., Lan L.T. (2018). Score-Based Fusion Schemes for Plant Identification from Multi-Organ Images. VNU J. Sci. Comput. Sci. Commun. Eng..

[22] Gao H., Gao X., Feng Q., Li W., Lu Z., Liang T. (2020). Approach to plant species identification in natural grasslands based on deep learning. Pratacultural Sci..

[23] Bisen D. (2021). Deep Convolutional Neural Network Based Plant Species Recognition through Features of Leaf. Multimed. Tools Appl..

[24] Hussain A., Barua B., Osman A., Abozariba R., Asyhari A.T. Performance of MobileNetV3 Transfer Learning on Handheld Device-Based Real-Time Tree Species Identification. Proceedings of the 2021 26th International Conference on Automation and Computing (ICAC).

[25] Hassan S.M., Maji A.K., Jasiński M., Leonowicz Z., Jasińska E. (2021). Identification of Plant-Leaf Diseases Using CNN and Transfer-Learning Approach. Electronics.

[26] Arshed M.A., Ghassan H., Hussain M., Hassan M., Kanwal A., Fayyaz R. A Light Weight Deep Learning Model for Real World Plant Identification. Proceedings of the 2022 Second International Conference on Distributed Computing and High Performance Computing (DCHPC).

[27] Guang J., Xi Z. (2022). ECAENet: EfficientNet with Efficient Channel Attention for Plant Species Recognition. J. Intell. Fuzzy Syst. Appl. Eng. Technol..

[28] Li C., Yao A. (2023). KernelWarehouse: Towards Parameter-Efficient Dynamic Convolution 2023. arXiv.

[29] Misra D., Nalamada T., Arasanipalai A.U., Hou Q. Rotate to Attend: Convolutional Triplet Attention Module 2020. Proceedings of the IEEE/CVF Winter Conference on Applications of Computer Vision.

[30] Dai X., Chen Y., Xiao B., Chen D., Liu M., Yuan L., Zhang L. Dynamic Head: Unifying Object Detection Heads with Attentions 2021. Proceedings of the IEEE/CVF Conference on Computer Vision and Pattern Recognition.

[31] Mwitta C., Rains G.C., Prostko E. (2024). Evaluation of Inference Performance of Deep Learning Models for Real-Time Weed Detection in an Embedded Computer. Sensors.

[32] Li Y., Ma C., Li L., Wang R., Liu Z., Sun Z. (2024). Lightweight Tunnel Obstacle Detection Based on Improved YOLOv5. Sensors.

[33] Gallo I., Rehman A.U., Dehkordi R.H., Landro N., La Grassa R., Boschetti M. (2023). Deep Object Detection of Crop Weeds: Performance of YOLOv7 on a Real Case Dataset from UAV Images. Remote Sens..

[34] Wang Q., Zhang Z., Chen Q., Zhang J., Kang S. (2024). Lightweight Transmission Line Fault Detection Method Based on Leaner YOLOv7-Tiny. Sensors.

